# NOD-Like Receptors: Master Regulators of Inflammation and Cancer

**DOI:** 10.3389/fimmu.2014.00327

**Published:** 2014-07-14

**Authors:** Mansi Saxena, Garabet Yeretssian

**Affiliations:** ^1^Department of Medicine, Immunology Institute, Icahn School of Medicine at Mount Sinai, New York, NY, USA; ^2^Tisch Cancer Institute, Icahn School of Medicine at Mount Sinai, New York, NY, USA

**Keywords:** apoptosis, autophagy, colorectal cancer, innate immunity, intestinal inflammation, inflammasome, nod-like receptors, nodosome

## Abstract

Cytosolic NOD-like receptors (NLRs) have been associated with human diseases including infections, cancer, and autoimmune and inflammatory disorders. These innate immune pattern recognition molecules are essential for controlling inflammatory mechanisms through induction of cytokines, chemokines, and anti-microbial genes. Upon activation, some NLRs form multi-protein complexes called inflammasomes, while others orchestrate caspase-independent nuclear factor kappa B (NF-κB) and mitogen activated protein kinase (MAPK) signaling. Moreover, NLRs and their downstream signaling components engage in an intricate crosstalk with cell death and autophagy pathways, both critical processes for cancer development. Recently, increasing evidence has extended the concept that chronic inflammation caused by abberant NLR signaling is a powerful driver of carcinogenesis, where it abets genetic mutations, tumor growth, and progression. In this review, we explore the rapidly expanding area of research regarding the expression and functions of NLRs in different types of cancers. Furthermore, we particularly focus on how maintaining tissue homeostasis and regulating tissue repair may provide a logical platform for understanding the liaisons between the NLR-driven inflammatory responses and cancer. Finally, we outline novel therapeutic approaches that target NLR signaling and speculate how these could be developed as potential pharmaceutical alternatives for cancer treatment.

## Introduction

Over the past two decades, immunologists have begun to appreciate the complexity of the innate immune system, its importance as the first wave of defensive action against perceived harmful microbes or foreign particles and its functions in triggering antigen-specific responses by engaging the adaptive immune system. Innate immune responses are orchestrated by germline-encoded pattern recognition receptors (PRRs) (1). PRRs recognize conserved pathogen-derived and damaged self-derived molecular components, commonly referred to as pathogen associated molecular patterns (PAMPs) and danger associated molecular patterns (DAMPs), respectively (2, 3). PRR superfamilies are broadly classified based upon structural homology and the requirement of different adaptor proteins that ensure their function and downstream signal transduction (4). The PRRs include members of the Toll-like receptors (TLRs) (3), nucleotide-binding, and oligomerization domain containing receptors [NOD-like receptors (NLRs)] (5, 6), retinoic acid-inducible gene (RIG) I-like RNA helicases (7), C-type lectins (8), and AIM2 like receptors (ALRs) (9). Evidence in the field points to a paramount importance of NLRs in human diseases with increasing interest in translating this knowledge toward clinical benefits. Due to the active role of NLRs in regulating pro-inflammatory signals and recruiting the adaptive arm of the immune system, dysregulation of microbial sensing has been reported to influence disease outcomes and tumorigenesis (10). In this review, we will describe the crucial roles of NLRs in cancer development and progression, and discuss the possibility of NLRs as targets for tumor therapy.

## Factors That Influence Tumorigenesis

Observations by Rudolf Virchow in the nineteenth century indicated a link between inflammation and cancer, and suggested that immune and inflammatory cells are frequently present within tumors. Indeed, chronic inflammation plays critical roles in various stages of cancer development and progression (11–13). Many cancer risk factors are associated with a source of inflammation or act through inflammatory mechanisms such as those evoked by bacterial and viral infections (14), tobacco smoke (15), obesity (16, 17), and aging or cell senescence (18, 19). While some cancers arise from chronic inflammation or after immune deregulation and autoimmunity, solid malignancies elicit intrinsic immune mechanisms that guide the construction of a tumorigenic microenvironment (12, 13, 20). Although the exact mechanism of how inflammation leads to neoplastic transformation is not fully known, it is suggested that inflammatory immune cells like macrophages and T cells are the main orchestrators of inflammation-mediated tumor progression. These cells secrete cytokines and chemokines that cause DNA damage, generate mutagenic reactive oxygen species (ROS), and supply cancer cells with growth factors (13). In addition, inflammatory mechanisms were shown to promote genetic instability by impairing DNA repair mechanisms, altering cell cycle checkpoints, and often facilitating epigenetic silencing of anti-tumor genes, thus contributing to the high degree of genetic heterogeneity in tumors (21). Oncogenic mutations prompted by an inflammatory microenvironment frequently cause neoplastic transformation by promoting excessive proliferation and resistance to cell death (22). Indeed, impaired expression and activity of proteins that control cell survival, such as the inhibitor of apoptosis proteins (IAPs) and the BCL2 family of proteins, is a common occurrence in many cancers (23, 24). Typically known to exert strong anti-apoptotic functions, IAPs neutralize pro-apoptotic second mitochondrial activator of caspases (SMAC) and inhibit activation of apoptotic caspases, thereby promoting cell survival during both physiological stresses and pathogenic stimulations (25–29). Owing to their strong pro-survival potency, enhanced expression of IAPs has been correlated with several human cancers (22). Unlike IAPs, the BCL2 family of proteins consists of both pro- and anti-apoptotic proteins that control critical checkpoints of intrinsic apoptosis by regulating mitochondrial integrity and release of cytochrome *c* into the cytosol (30). Deregulation of the functions of BCL2 proteins, i.e., down-regulation of pro-apoptotic members and over expression of pro-survival members, has been strongly correlated with tumorigenesis and resistance to chemotherapy (31). Interestingly, the pro-apoptotic BID, PUMA, and NOXA are transcriptional targets of the tumor suppressor gene p53 and loss of their expression enhances tumorigenesis and morbidity of MYC overexpressing transgenic mice (32, 33). It was described that the transcription factor p53 senses physiological stresses and is critical for restraining tumor growth. Indeed, loss of p53 expression or function in both humans and mice has been proven to promote sporadic tumorigenesis (34, 35). Induction of target genes that inhibit cancer progression is generally considered to be the canonical mechanism of p53-mediated tumor-suppression. These target genes directly modulate cellular programs involving induction of apoptosis, cell cycle arrest, and promotion of cellular senescence and DNA repair (36). Recently, non-canonical functions of p53 have come to light, like the regulation of cellular metabolism, cell-to-cell communication, autophagy, tumor invasion, and metastasis, making p53 an attractive pharmaceutical target for treating cancers [reviewed in Ref. (37)]. Early detection of rogue tumor cells by the innate immune cells and their rapid removal is a key host defense strategy for evading tumorigenesis. In particular, natural killer (NK) cells are primary sentinels that guarantee such immune surveillance by differentiating normal cells from stressed or tumor cells via the expression of specific NK receptors (38). Indeed, increased presence of NK cells at tumor sites has been reported to improve remission, whereas decreased NK cell anti-tumor activity has been correlated with a greater likelihood for developing cancer (39).

## Nod-Like Receptors in Cancer

### Overview of NLRs

**Figure 1:**
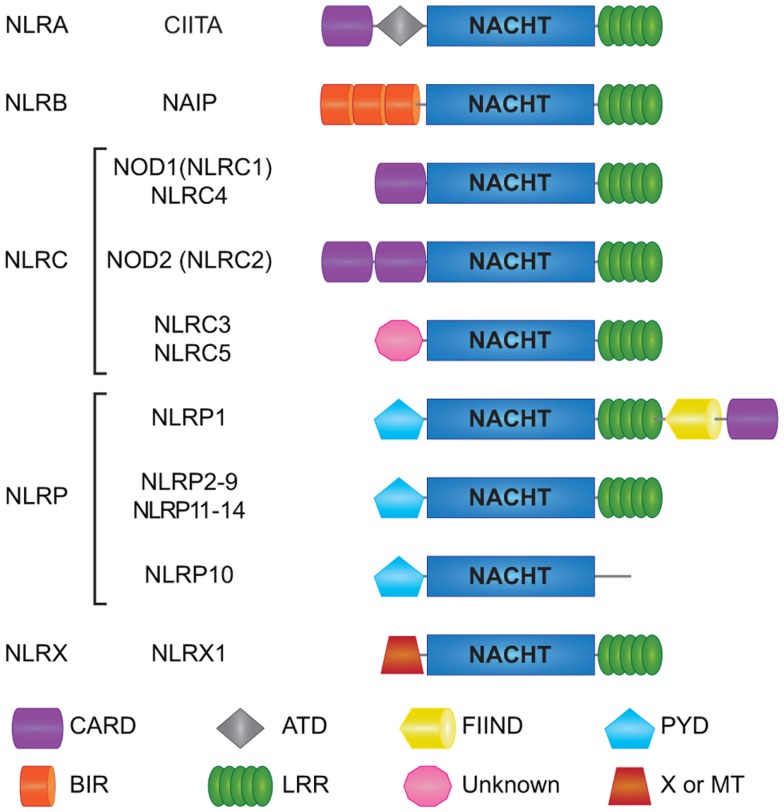
**Schematic representation of individual NLR domains**. Domain architecture of human NLRs is depicted here. Human NLRs are sub-classified into five categories: NLRA, NLRB, NLRC, NLRP, and NLRX. All 22 human NLRs contain a central NACHT domain and a C-terminal ligand sensing LRR domain, with the exception of NLRP10. The N-terminal domains ascribe functional properties to the NLRs; however, the function of some of the domains is still unclear like for the N-terminal domain of NLRC3 and NLRC5, as well as the C-terminal FIIND in NLRP1. CARD; caspase association and recruitment domain, ATD; acidic transactivation domain, FIIND; function to find domain, PYD; pyrin domain, BIR; Baculoviral inhibition of apoptosis protein repeat domain, LRR; leucine-rich repeats, MT; targets NLRX1 to the mitochondria but no sequence homology with traditional mitochondrial targeting sequence has been reported.

NOD-like receptors are a relatively recent addition to the PRR superfamily (40–42). All NLRs contain a central NACHT domain that facilitates oligomerization, and bear multiple leucine-rich repeats (LRRs) on their C-terminal for ligand sensing (5, 43). The 22 human NLRs can be distinguished into five subfamilies by their N-terminal effector domains that bestow unique functional characteristics to each NLR (43) (Figure 1). NLRs with an N-terminal acidic transactivation domain are termed NLRA (CIITA) and serve as transcriptional regulators of MHC class II antigen presentation (44). NLRB (NAIP) proteins have an N-terminal baculoviral inhibition of apoptosis repeat (BIR) domain and are largely recognized for their roles in host defense and cell survival. For instance, NAIP5 is known to induce host defense against bacterial infections by curtailing macrophage permissiveness to *Legionella pneumophila*, the causative agent of the Legionnaires’ disease (45–47). N-terminal caspase activation and recruitment domain (CARD) distinguishes the NLRC subfamily (NLRC 1–5) and allows direct interaction between members of this family and other CARD carrying adaptor proteins. NOD1 (NLRC1) and NOD2 (NLRC2), the founding members of the NLRs, are key sensors of bacterial peptidoglycan (PGN) and are crucial for tissue homeostasis and host defense against bacterial pathogens (48). Notably, single-nucleotide polymorphisms (SNPs) in the *NOD2* (*CARD15*) gene are among the most significant genetic risk factors associated with Crohn’s disease (CD) susceptibility (49, 50), hence the rising interest in unraveling the functions of NOD1 and NOD2 receptors in microbial sensing, intestinal homeostasis, and disease. Members of the pyrin domain (PYD) containing NLRP subfamily (NLRP 1–14) are best known for their role in inducing the formation of the oligomeric inflammatory complex “Inflammasome” (51). NLRX1, the only described member of the NLRX subfamily contains an N-terminal mitochondria-targeting sequence required for its trafficking to the mitochondrial membrane (Figure 1). Mechanistically, NLRX1 was shown to down-regulate mitochondrial anti-viral signaling protein (MAVS)-mediated type I interferon (IFN) production (52), interfere with the TLR-TRAF6-NF-κB pathways (53, 54), and enhance virus induced-autophagy (55, 56). On the other hand, NLRX1 was implicated in the generation of ROS induced by TNFα and Shigella infection magnifying the JNK and NF-κB signaling (57). Interestingly, NLRX1-mediated ROS generation was involved in promoting *Chlamydia trachomatis* replication in epithelial cells (58). However, recent data from Soares et al. revealed that bone marrow macrophages (BMMs) and mouse embryonic fibroblasts (MEFs) from Wild type (WT) or *Nlrx1*^−/−^ mice respond equally to *in vitro* infection with Sendai virus or following *in vivo* challenge with influenza A virus and TLR3 ligand Poly I:C (59). Additionally, Rebsamen et al. reported no significant contribution of NLRX1 in RLR–MAVS signaling both *in vitro* and *in vivo* (60). Overall, the precise role of NLRX1 remains controversial and further research is required to validate its pro or anti-inflammatory properties.

Dysregulated apoptosis and autophagy pathways, as well as excessive chronic inflammation are major drivers of carcinogenesis. NLRs are innate immune sensors that actively communicate with a myriad of cell death regulators. Hence, these PRRs are well-positioned to influence tumor development and progression particularly at sites with high host-microbiome interactions like the gut. One of the mysteries of the innate immune system is how do NLRs sense molecular patterns from both commensal and pathogenic microorganisms and manage to tolerate one while help eradicate the other (5, 61). This disparity in NLR functions is particularly useful in the intestinal epithelia where host cells are in constant contact with millions of microbes. Consequently, it came as little surprise when common variants in the NLR genes were correlated with the incidence of CD and susceptibility to cancers (50, 62–64). Due to these correlations, most of the studies have been focused on understanding the mechanisms by which NODs and inflammasome NLRs regulate intestinal inflammation and tumorigenesis.

## NOD1 and NOD2 in Cancer

### NOD-dependent signaling cascades

**Figure 2:**
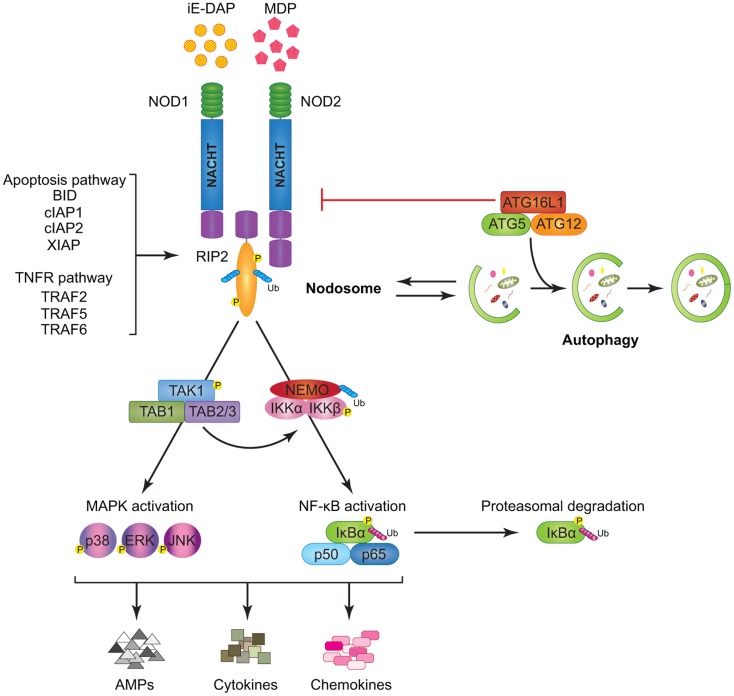
**Model of NOD1 and NOD2 signaling cascades**. NOD1 and NOD2 recognize bacterial PGNs, iE-DAP, and MDP, respectively. Following ligand sensing the NODs recruit their common adaptor RIP2 by CARD–CARD interactions and induce RIP2 to undergo phosphorylation. The members of the TRAF family (TRAF2, TRAF5, and TRAF6), the IAP family (XIAP, cIAP1, and cIAP2), and the BCL2 family (BID) bind to RIP2 and facilitate its ubiquitination allowing the recruitment of TAK1 and ubiquitinated NEMO to the Nodosome. On one hand, NEMO instigates activation of the canonical NF-κB pathway by phosphorylating IKKα and IKKβ, by inducing IκBα phosphorylation and proteasomal degradation, and by freeing p50 and p65 NF-κB subunits. On the other hand, TAK1 recruits TAB1 and TAB2/3 inducing both (p38, ERK, and JNK) MAPK and NF-κB activation. Stimulation of both arms culminates in the induction of anti-microbial peptides (AMPs), cytokines, and chemokines. The formation of the Nodosome promotes autophagy and conversely, a fully functional autophagy machinery helps in signal transduction through the Nodosome. ATG16L1 along with ATG5 and ATG12 is required for autophagosome formation, however, independently of its autophagy functions, ATG16L1 negatively regulates NOD/RIP2 signaling.

NOD1 and NOD2 are cytosolic proteins that sense intracellular bacterial PGN and trigger signal transduction via NF-κB and MAPK activation. NOD1 is expressed in both hematopoietic and non-hematopoietic cells and responds to intracellular gamma-d-glutamyl-meso-diaminopimelic acid (iE-DAP) mostly present on Gram-negative bacteria and only on some select Gram-positive bacteria, like *Listeria* and *Bacillus* species (65–67). Unlike NOD1, NOD2 expression is largely restricted to hematopoietic cells and certain specialized epithelial cells such as the small intestinal Paneth cells (68). NOD2 recognizes cytosolic muramyl dipeptide (MDP) found in the PGN of all bacteria (69). Besides providing immunity against intracellular bacteria, NODs were revealed to be critical for host defense against non-invasive Gram-negative bacteria like *Helicobacter pylori*, following delivery of its PGN into the host cells through the bacterial type IV secretion system (70). Moreover, NOD1 and NOD2 ligands were also described to gain access to the cytosol by endocytosis with the help of transporter proteins like SLC15A3 and SLC15A4 (71–73). Notably, NOD1 and NOD2 have been reported to localize to the plasma membrane at the sites of infection; however, the biological relevance of this translocation remains elusive (74, 75). Interestingly, a recent report accentuated the importance of NOD proteins in monitoring the activation state of small Rho GTPases (e.g., RAC1, CDC42, and RHOA) and inducing unusual immune responses in the host in response to bacterial infection (76). Upon activation by their cognate ligands both NOD1 and NOD2 self-oligomerize, undergo a conformational change, and through homotypic CARD–CARD interactions allow the recruitment of the CARD containing adaptor Receptor-interacting protein kinase 2 (RIP2 or RIPK2) (41, 42, 77, 78) (Figure 2). This event facilitates the formation of a multi-protein signaling complex termed “Nodosome,” which leads to downstream NF-κB and MAPK-mediated inflammatory and anti-microbial output. Indeed, cells or mice lacking RIP2 do not respond to NOD agonists and fail to produce pro-inflammatory and anti-microbial molecules (78–80). Initially, it was thought that NOD oligomerization initiated RIP2 aggregation and activation by “induced proximity” (81). While this model still stands true, over the years new body of research has contributed a wealth of data regarding specific sequence of events that leads to RIP2 activation. In contrast to the earlier studies (82–85), recent *in vitro* data using pharmacological inhibitors as well as *in vivo* evidence using a knock-in mouse with kinase-dead RIP2 (K47A) have highlighted the key role of the kinase activity of RIP2 in NOD-mediated immune responses (86, 87).

Lately, it was described that the pathways activated downstream of NOD proteins are closely related to those activated by death receptors, notably TNF receptor 1 (TNFR1). For instance, hierarchical recruitment of selective TNFR-associated factors (TRAF2, TRAF5, or TRAF6) facilitates Lys63 poly-ubiquitination and activation of RIP2 (88–90). Activated RIP2 facilitates ubiquitination of NEMO (also called IKKγ) leading to the recruitment of tumor growth factor β-activated kinase 1 (TAK1) and TAK1 binding proteins (TAB) 1, TAB2, or TAB3 (91, 92). Following this complex formation, IKKs (IKKα and IKKβ) get phosphorylated eventually driving the phosphorylation and degradation of IκBα and subsequent transcription of NF-κB target genes (5, 89, 92) (Figure 2). RIP2 activation also constitutes a key event that links the NOD–RIP2 cascade with the p38, extracellular signal-regulated kinase (ERK), and c-Jun N-terminal kinase (JNK) MAPK pathways (93).

In addition to TRAFs, members of the IAP family including X-linked IAP (XIAP) and cellular IAP1 (cIAP1) and cIAP2 were described to physically interact with RIP2 and facilitate NOD-mediated immunity (94–98). Both *in vitro* and *in vivo* studies suggest a strong role for cIAP1 and cIAP2 in promoting NOD signaling (Figure 2); however, the mechanism for such positive regulation is still not fully understood (94, 99–101). Similarly, XIAP was reported to recruit a linear ubiquitin chain assembly complex (LUBAC) for RIP2 ubiquitination and this step was proven critical for downstream NF-κB regulation (96, 97). Upon microbial sensing another E3 ubiquitin ligase, ITCH, also ubiquitinates RIP2, and it is speculated that ITCH-mediated ubiquitination acts like a molecular switch dictating the fate of the signaling circuit to NF-κB or p38 and JNK activation (102). Pathogen-mediated NOD1 activation has also been shown to elicit protective immune responses via RIP2-TRAF3-IRF7-mediated transcription of IFNβ (79). Overall, it is tempting to speculate that similar to pro-survival association of RIP1 with cIAP1 and cIAP2 (103), interactions between RIP2 and the IAPs may also lead to modulation of cellular apoptosis. However, neither NODs nor RIP2 has been demonstrated to exploit these associations to affect cell survival. Similarly, several studies have alluded to NODs as being regulators of caspase-mediated apoptosis (82, 104, 105); yet, no direct link has so far been reported. Recently, the pro-apoptotic BH3 only BCL2 family protein BID (BH3 interacting-domain death agonist) was identified in a genome wide siRNA screen as a positive regulator of NOD signaling (101). BID was demonstrated to bind to RIP2 bridging both NOD and IKK complexes to specifically transduce NF-κB and ERK signaling events (101). Notably, BID was phosphorylated upon activation with NOD agonists and these innate immune functions of BID were found to be independent of its pro-apoptotic processing by caspase-8 (101). The discovery involving a classical pro-apoptotic protein, such as BID, in NOD–RIP2 signaling strengthens the concept that inflammatory and cell death pathways do not function as discrete mechanisms but share common adaptors. Such adaptors can exert multiple functions depending upon the nature of the stimuli (5, 106–108) (Figure 2). One recent study have reported that BID-deficient mice exhibit a normal NOD-mediated immunity (109), suggesting that further investigations are still needed to clearly decipher the implication of BID in NOD signaling.

Similar to *NOD2*, a SNP encoding a missense variant in the autophagy gene *ATG16L1* was strongly associated with the incidence of CD, raising a possible common role of both genes in host defense mechanisms (110, 111). Intriguingly, it has been described that NOD1 and NOD2 stimulation enhances autophagy, either directly by interacting with ATG16L1 (112) or indirectly (112–115). Conversely, pharmacological inhibition of both early and late autophagy has been proven to down-regulate MDP-mediated NF-κB and MAPK activation, suggesting that autophagocytic trafficking of MDP may be required for efficient NOD2 signaling (114). Surprisingly, ATG16L1 was recently shown to negatively regulate NOD1- and NOD2-mediated inflammatory signaling by interfering with RIP2 ubiquitination and recruitment to the Nodosome (116) (Figure 2). Taken together, this information suggests that different NLRs can have opposing regulatory effects on autophagy and cell death, yet the molecular triggers that dictate these actions are not fully understood.

## NOD Proteins and Cancer

Three mutations within the LRR region of the *NOD2* gene have been associated with increased CD susceptibility. Interestingly, these same mutations have also been found to directly interfere with NOD2’s bacterial sensing faculties and downstream NF-κB activation (49, 50). Notably, such inactivation of NOD2 immunity has been indicated to enhance the risk of bacterial infections following chemotherapy in patients with acute myeloid leukemia (117). In addition, *NOD2* polymorphisms have been correlated with modifications in gastric mucosa and increased risk for *H. pylori* induced gastric cancer (118). Apart from intestinal disorders, mutations in NOD2 have been linked with increased prevalence of early onset breast (119) and lung cancers (120, 121). However, how NOD2 contributes to the initiation and the progression of cancer remains ill defined. Although no mutations in the *NOD1* gene have been so far associated with the incidence of intestinal inflammation or even colorectal cancer (CRC), murine models clearly designate a central anti-tumorigenic function for NOD1 in the pathophysiology of disease. For instance, *Nod1*^−/−^ mice have been described to be susceptible to dextran sulfate sodium (DSS), a sulfated polysaccharide highly toxic to enterocytes (122). Upon combination of a single hit of the carcinogen, azoxymethane (AOM), with DSS (123), NOD1-deficient mice were found to develop significantly more and larger colonic tumors as compared to WT mice (122). This experimental CRC model is particularly applicable when the focus is on understanding colitis-driven tumor initiation and progression. The *Apc^Min/^*^+^ mouse is a *N*-Ethyl-*N*-Nitrosourea (ENU) mutant model carrying the multiple intestinal neoplasia (Min/+) mutation and recapitulates many aspects of human hereditary or sporadic CRCs with mutations in the adenomatous polyposis coli (Apc) gene (124–127). Intriguingly, it has been reported that treatment with low doses of DSS leads to increased colonic tumors in *Apc^Min/^*^+^*Nod1*^−/−^mice suggesting that NOD1 serves as a negative regulator of the tumor-promoting Wnt/β-catenin cascade (128, 129). Further analysis revealed that absence of NOD1 exacerbated NF-κB-mediated inflammation early during colitis causing gut barrier damage and prompted a second wave of microbiota driven inflammation and intestinal epithelial cell (IEC) proliferation, thus initiating tumor development. These conclusions are supported by the observation that antibiotic treatment of *Nod1*^−/−^ mice ameliorated DSS-induced CRC (122). While most investigations have been focused on the role of NOD1 in models of intestinal tumorigenesis, one report provided experimental evidence for the protective role of NOD1 in breast cancer (104). Herein, it was shown that NOD1-deficient MCF-7 breast cancer cells were resistant to iE-DAP and cycloheximide mediated cell death. Interestingly, SCID mice grafted with NOD1 overexpressing cells exhibited rapid tumor regression. In sharp contrast, mice grafted with NOD1-deficient MCF-7 cells displayed increased and continued tumor growth (104).

Like *Nod1*^−/−^ mice, NOD2-deficient mice have been revealed to be highly susceptible to DSS-induced colitis by inheritance of dysbiotic microbiota that markedly sensitizes mice to injury (130). Furthermore, *Nod2*^−/−^ mice have been found to display worse disease outcome with increased epithelial dysplasia, heightened tumor burden, and elevated expression of the pro-inflammatory cytokine IL-6 when subjected to AOM–DSS treatment. This transmissible phenotype was significantly ameliorated upon treatment with broad-spectrum antibiotics or using the neutralizing IL-6 receptor antibody (130). Altogether, these findings reinforce the idea that aberrant NOD signaling gives rise to dysbiosis that in an inflammatory setting ultimately causes mucosal injury and drives CRC. So far, the translational value of this knowledge is limited but with the recent technological advances in the microbiome research it is predicted that modulation of dysbiosis could be used as a therapeutic strategy for patients with CD as well as CRC.

Contrary to the protective role for NODs in intestinal tumorigenesis, increased expression of both NOD1 and NOD2 has been reported in the head and neck squamous cell carcinoma biopsies as compared to the healthy nasal biopsies. These findings implicate NODs in enhancing head and neck cancers; however, thus far no corroborating experimental evidence has been reported (131). Furthermore, iE-DAP stimulation of human pharyngeal squamous carcinoma cell lines (Detroit 562 and Fadu) has been determined to augment the production of β-defensins, which can serve as chemoattractants, thus fostering an inflammatory and pro-tumorigenic environment (131).

## Inflammasome NLRs in Cancer

### Inflammasome NLRs: NLRP3-mediated signaling cascades

**Figure 3:**
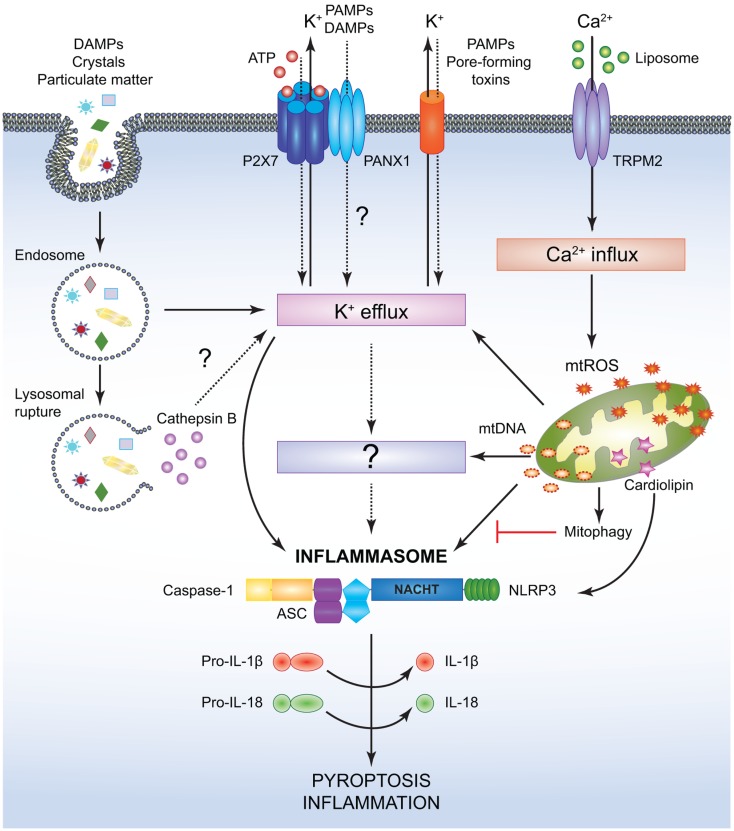
**Simplified mechanisms for the canonical NLRP3- inflammasome activation**. Various PAMPs and DAMPs provide the signal 2 required to assemble and activate the NLRP3 inflammasome comprised of NLRP3, ASC, and caspase-1. Although the precise mechanism leading to NLRP3 activation is still controversial, it is speculated that K^+^ efflux may be the common cellular response that triggers inflammasome activation. However, this notion has not been fully verified and it is possible that an unidentified or intermediate adaptor may be required for transmitting signals between K^+^ efflux and the NLRP3 inflammasome. Crystals and particulate DAMPs enter the cell via endocytosis directly inducing K^+^ efflux and NLRP3-inflammasome formation. In addition, the endo- lysosomes carrying these DAMPs undergo lysosomal rupture and release cathepsin B, which acts as an intracellular DAMP and can induce K^+^ efflux. However, contradicting studies indicate that lysosomal rupture may cause K^+^ efflux and inflammasome activation even in the absence of cathepsin B. ATP binds to the P2X7 receptor on the cell membrane and causes opening of the PANX1 channels allowing K^+^ efflux and influx of any PAMPs and DAMPs present in the extracellular space. PAMPs such as pore-forming toxins activate the NLRP3 inflammasome and facilitate K^+^ efflux. Liposomes instigate Ca^2+^ influx through opening of the TRPM2 channels. Accumulation of excessive Ca^2+^ in the cytosol causes mitochondrial dysfunction and release of mtROS and oxidized mtDNA, which may activate the NLRP3 inflammasome either directly or by inducing K^+^ efflux. Clearance of distressed mitochondria by mitophagy serves to evade such inflammasome activation. Mitochondrial Cardiolipin binds to NLRP3 and is required for the NLRP3-inflammasome activation. Following NLRP3-inflammasome assembly, caspase-1 undergoes proximity driven proteolytic cleavage and further processes pro-IL-18 and pro-IL-1β into their mature active forms. Activation of the NLRP3-caspase-1 axis results in inflammation and pyroptotic cell death.

While NOD1 and NOD2 form the Nodosome, other NLRs assemble macromolecular inflammasome complexes. To date, various inflammasome platforms have been described (132), but the NLRP3 inflammasome is the most commonly studied. The reason behind this could be the initial discovery of mutations in the *NLRP3* (*CIAS1*) gene implicating this PYD containing protein in both familial cold auto-inflammatory syndrome (FCAS) and Muckle–Wells Syndrome (MWS) (133). Thus, the NLRP3 inflammasome will be described here as a prototype for these NLRs (Figure 3). Classically, the inflammasome has been described to consist of an NLRP, the inflammatory protease caspase-1, and the apoptosis-associated speck like protein (ASC) (51). ASC contains an N-terminal PYD and a C-terminal CARD making it uniquely suited for bringing into close proximity the two key components, caspase-1 and NLRPs (134, 135). Upon activation, NLRP3 recruits ASC and caspase-1, which is a prerequisite for the cleavage and maturation of the inflammatory cytokines IL-1β and IL-18 and consequent inflammatory cell death named pyroptosis (136–141). Lately, a more complex model for NLRP3-inflammasome activation has been proposed where two adaptors, ASC and mitochondrial MAVS, are required for optimal inflammasome triggering (142).

Owing to its widespread expression in numerous cell types such as neutrophils, monocytes, DCs, epithelial cells, and T cells (140, 143, 144), NLRP3 is exposed to a wide array of PAMPs and DAMPs that instigate the assembly and activation of the inflammasome [reviewed in Ref. (5, 132, 145–148)]. The NLRP3-inflammasome formation requires a two-step process (149). The priming step (or signal 1) involves TLR-NF-κB-driven induction of inflammasome components, as basal expression of NLRP3 in resting cells is insufficient for effective inflammasome activation (149, 150). However, certain cells like the human blood monocytes and murine macrophages appear to activate the NLRP3 inflammasome in response to LPS stimulation alone (151, 152). It is noteworthy that a transcriptionally silent mechanism for TLR4-mediated inflammasome priming has been lately discovered (153, 154). This mechanism involves mitochondrial ROS (mtROS)-driven deubiquitination of NLRP3, suggesting that constitutive ubiquitination of NLRs may be a homeostatic mechanism to prevent overt inflammasome activity (154). The second activation step (or signal 2) promotes the NLRs to undergo homotypic oligomerization and assemble the inflammasome.

While several models have been proposed to define the signals behind NLRP3 activation, the precise mechanisms remain hitherto unresolved. Various bacterial pathogens induce potassium efflux and activate the NLRP3 inflammasome via the action of secreted pore-forming toxins (e.g., nigericin from *Streptomyces hygroscopicus*, listeriolysin O from *Listeria monocytogenes*, pneumolysin from *Streptococcus pneumoniiae*, alpha-hemolysin, etc.) (138, 155, 156) (Figure 3). In addition, NLRP3 inflammasomes have been known to assemble in response to cytosolic bacterial and viral RNA both *in vivo* and *in vitro* (137, 157–160). Extracellular adenosine tri-phosphate (ATP) released from dying or damaged cells also causes NLRP3-inflammasome activation through either paracrine or autocrine sensing of ATP by the purinergic receptor P2X7 (138, 161–163). Besides, it has been defined that ATP released from phagocytosed dying cells acts similarly on P2X7 and prompts pannexin-1 (PANX1) channels to open, thus resulting in potassium (K^+^) efflux and allowing other agonists to further engage and activate NLRP3 (164) (Figure 3).

Monosodium urate (MSU) and calcium pyrophosphate dehydrate crystals, alum, amyloid-β fibrils, as well as environmental pollutants like asbestos and silica strongly activate the NLRP3 inflammasome (139, 165–170). According to one model for this mode of activation, uptake of the crystalline and particulate matters into the cell causes lysosomal destabilization and release of cathepsin B, which is sensed by NLRP3 (168, 169). Interestingly, however, opposing results were obtained when cathepsin B-deficient BMMs were used to test this hypothesis, as no differences in IL-1β or caspase-1 cleavage were observed in response to several inflammasome activators such as hemozoin, MSU, or alum (171). Another model suggests that these activators prompt generation of mtROS and mitochondrial DNA, both of which are responsible for NLRP3-inflammasome activation (172–174). Evidently, pharmacological inhibition of mtROS production has been shown to prevent NLRP3-inflammasome formation indicating that ROS generation is an upstream event for NLRP3 activation (165, 166) (Figure 3). Liposomes have been found to induce mtROS and NLRP3-inflammasome activation by triggering calcium (Ca^2+^) influx via the transient receptor potential melastatin 2 (TRPM2), although the exact mechanism linking ROS production to TRPM2 channel opening is still not well-characterized (175). On the other hand, the mitochondrial protein cardiolipin has been shown to directly bind and activate NLRP3 in a ROS-independent manner suggesting that ROS may not be the common denominator engaging the NLRP3 inflammasome (176). Recent advances have put forward additional mechanisms underlying NLRP3-inflammasome activation. In BMMs stimulated with PAMPs, extracellular calcium has been shown to activate the calcium sensing receptor (CASR) mediating signal transduction pathways that culminate in the release of calcium stores from the endoplasmic reticulum (ER), eventually activating the NLRP3 inflammasome (177–179). The diverse nature of the NLRP3-inflammasome agonists allude to the likelihood that, instead of directly sensing PAMPs and DAMPs, NLRP3 may be activated by converging pathways with a final common ligand for NLRP3. Guanylate binding protein 5 (GBP5) has been recently proposed as one such component that directly participates in NLRP3-inflammasome activation; however, further investigation is needed to decipher how the GBP5 is activated and why it is required for select inflammasome assembly (180). Finally, studies by Munoz-Planillo et al. suggest that potassium efflux may perhaps be the sole intracellular event necessary for NLRP3 activation in response to a wide array of stimuli arguing for a unifying model for the NLRP3-inflammasome activation (181) (Figure 3).

Production of mtROS often culminates in mitophagy, an autophagic clearance of dysfunctional mitochondria. It has been demonstrated that inhibition of mitophagy enhances NLRP3-caspase-1-mediated secretion of IL-1β and IL-18 in response to LPS and ATP (172). In addition, deletion of ATG16L1 was found to promote IL-1β release in response to ATP, MSU, or LPS alone (182). Moreover, it has been recently suggested that autophagy may restrict NLRP3 activity by directly sequestering and targeting inflammasome components for degradation (183, 184). Overall, it is reasonable to speculate that autophagy could serve as a mechanism for preventing excessive NLRP3-inflammasome activation (172, 173, 183–185).

Mitochondrial dysfunction plays a central role in regulating the mechanisms involved in both inflammasome and apoptosis pathways. Loss of mitochondrial membrane potential is a pivotal event in intrinsic apoptosis and is tightly regulated by the BCL2 family of proteins through a system of checks and balances (30). Interestingly, anti-apoptotic BCL2 and BCL-XL proteins have been reported to directly interact with NLRP1 (CARD and PYD domain containing NLRP) to negatively regulate caspase-1 activation (186, 187). Similarly, BCL2 overexpression was shown to limit NLRP3-inflammasome activation (173, 174). In addition to BCL2 proteins, cIAP1, cIAP2, and XIAP have also been linked with inflammasome activation. Unlike their role in NOD signaling, initial studies have proposed that expression of these proteins might prevent caspase-1-dependent cell death (188). However, more recently cIAP1 and cIAP2 along with TRAF2 were found to enhance inflammasome activation seemingly by ubiquitinating and stabilizing caspase-1 and consequently prompting IL-1β release (189). In another report, genetic ablation of cIAP1 or cIAP2 had no effect on NLRP3-inflammasome activation, but concurrent pharmacological degradation of XIAP, cIAP1, and cIAP2 using SMAC mimetics was shown to limit caspase-1 activation (190). Interestingly, further inquiries revealed that in the absence of XIAP, cIAP1, and cIAP2, cell death in response to LPS was primarily incited by RIP3 activation causing NLRP3-caspase-1- as well as caspase-8-dependent IL-1β secretion (190). Lately, the concept of non-canonical inflammasome has been defined, which requires activation of caspase-11 in response to Gram-negative bacteria to facilitate either caspase-1-mediated IL-1β secretion or caspase-1-independent pyroptosis (191–194). Interestingly, apoptosis mediators FADD and caspase-8 have been involved in canonical and non-canonical NLRP3-inflammasome signaling. Indeed, FADD and caspase-8 facilitate the priming in “signal 1” by instigating both, LPS-TLR-MyD88-triggered induction of pro-IL-1β and NLRP3, as well as TLR-TRIF-mediated upregulation of pro-caspase-11 (195). Upon infection with *Citrobacter rodentium* or *Escherichia coli*, FADD and caspase-8 have been found to promote the “signal 2” by interacting with the NLRP3-inflammasome complex, thus influencing both canonical (caspase-1-dependent IL-1β maturation) and non-canonical (caspase-11-dependent pyropotosis) inflammasomes (194, 195). Conversely, it has been exhibited that caspase-8-deficient murine DCs are hyper-responsive to LPS-induced NLRP3-inflammasome assembly and activation (196). Overall, these studies place caspase-11 and caspase-8 at the center of inflammasome activation; however, a general lack of consensus in the field makes it hard to aptly judge their contribution in inflammasome-induced inflammation.

## Inflammasome NLRs and Cancer

*NLRP3*, previously associated with rare and severe auto-inflammatory disorders, has been lately implicated in CD susceptibility and correlated with decreased NLRP3 expression and IL-1β production (62). Indeed, mice lacking NLRP3 have been shown to display exacerbated colonic inflammation upon DSS-induced colitis characterized by greater gut barrier damage, inflammatory immune cell infiltration, and cytokine production (197, 198). In accord, a central role has been ascribed for caspase-1 and ASC in intestinal epithelial repair after DSS-injury (199). Specifically, caspase-1, ASC, or NLRP3 deficiency in mice has been shown to be detrimental in DSS-induced intestinal inflammation, a mechanism attributed to the lack of IL-18 production by IECs (198, 199). Concomitantly, the increased colitogenic phenotype was completely reversed when mice were exogenously administered with the recombinant IL-18 cytokine (198, 199). The same lack of inflammatory regulation was found to render *Nlrp3*^−/−^ and *Casp1*^−/−^ mice more susceptible to AOM–DSS carcinogenesis (197, 200). The heightened tumor growth in the caspase-1 deficient mice was accompanied with drastically low levels of colonic IL-18. Overall, NLRP3 was shown to be important for IL-18 secretion, which in turn through IFNγ production induces STAT1 (Signal transducers and activators of transcription 1) phosphorylation and thus promotes an anti-tumorigenic environment (200). Moreover, it has been shown that *Il18*^−/−^ or *Il18r*^−/−^ mice are more susceptible to DSS-induced colitis and CRC, mimicking the increased tumor burdens observed in NLRP3 and caspase-1 deficient mice (201). Recent findings have put forward a novel concept for the dual function of IL-18 in intestinal inflammation and colitis-driven CRC (202, 203). For instance, during acute injury IEC-derived IL-18 triggers repair and restitution of the ulcerated epithelial barrier, whereas under chronic inflammatory settings the excessive release of IL-18 both from IECs and lamina propria macrophages and DCs is deleterious (203, 204). A protective role for NLRP3 has also been described in hepatocellular carcinoma (HCC) (205). This correlation is primarily based on mRNA and protein expression data showing reduced levels of NLRP3 and other related inflammasome components seen in hepatic parenchymal cells derived from HCC tissue specimens as compared to non-cancerous liver sections (205). On the other hand, a gain of function SNP (Q705K) within the *NLRP3* gene has been associated with increased mortality in CRC patients (206). Significantly, the same SNP was also found to be more prevalent in patients with malignant melanoma (207). Human monocytic THP-1 cells overexpressing a mutant variant of NLRP3 bearing the Q705K SNP have been reported to greatly respond to the inflammasome agonist alum and to trigger the production of IL-1β and IL-18, implying that overt NLRP3 activation could be detrimental for certain types of cancer (208). Similarly, another group implicated constitutive NLRP3-inflammasome signaling in the development and progression of melanomas (209).

Loss of function in the tumor suppressor gene p53 has been associated with a large number of sporadic cancers (36). One of the mechanisms for p53-induced clearance of potentially carcinogenic cells has been found to be via transcriptional up regulation of cell death activators (210). In light of this knowledge, the discovery of NLRC4 as a downstream transcriptional target of p53 was a promising evidence for the anti-tumorigenic functions of this NLR (211). Moreover, lack of NLRC4 inflammasome has been associated with the attenuation of p53-mediated cell death, indicative of a protective role of NLRC4 during tumor development (211). Several groups have investigated the role of NLRC4 in colitis and CRC. However, lack of consensus in the susceptibility of *Nlrc4*^−/−^ mice to DSS as well as AOM–DSS treatment makes it difficult to gage the protective effect of NLRC4 in these models (197, 212). It has been demonstrated that mice deficient in NLRC4 develop higher tumor burdens than WT mice when subjected to DSS-induced CRC (212). In addition, bone marrow chimera experiments verified that NLRC4 expression within the radioresistant compartment was the major driver of CRC protection (212). Surprisingly, similar colitic phenotypes have been observed between WT and *Nlrc4*^−/−^ mice following DSS administration, suggesting that tumor regulation by NLRC4 is mostly cell intrinsic and not through down-regulation of inflammation (213). Given the unique capacity of NLRC4 to sense and differentiate between commensal and pathogenic microbes in the gut (214), it is surprising that the tumor restraining roles of NLRC4 have been ruled to be independent of its immune regulatory functions. One unifying theory addressing these discrepancies could be that anti-tumor functions of NLRC4 are attributed to the cells of non-hematopoietic origin, whereas intestinal mononuclear phagocytes are the primary source of NLRC4 for microbial sensing and pathogen clearance (213, 214). Overall, these assumptions warrant deeper inquiries to clearly elucidate the mechanisms by which NLRC4 exerts protective functions during CRC and to decipher the relevance of p53-mediated role of NLRC4 in tumorigenesis.

Akin to NLRP3, both NLRP6 and NLRP12 have been recently described to use ASC-caspase-1 molecular platforms and assemble inflammasomes. A first hint of NLRP6 being an inflammasome NLR was gleaned from *in vitro* experiments showing increased caspase-1 cleavage when ASC and NLRP6 were co-expressed (215). Further *in vivo* evidence emphasized a protective role for NLRP6 in intestinal inflammation and tumorigenesis as *Nlrp6*^−/−^ mice showed high susceptibility to DSS-induced colitis and AOM–DSS-induced CRC (216–218). Unlike NLRC4, dampening of inflammation is purported to be one of the primary mechanisms for NLRP6-mediated protection and tissue homeostasis. NLRP6 has been shown to promote a gut microbiome that limits chronic inflammation. In fact, it has been evidenced that *Nlrp6*^−/−^ mice display a distinct transmissible pro-colitogenic microbiome with increased prevalence of the bacterial genus *Prevotellaceae* (217). These mice presented a steady state colitic phenotype and an enhanced sensitivity to DSS colitis (217). Overall, a mechanism has been suggested wherein dysbiosis in the gut, caused by aberrant NLRP6 inflammasome signaling, drives excessive CCL5-mediated IL-6 production, barrier damage, and inflammation (217). In agreement with the findings in *Casp1*^−/−^ mice (199), NLRP6-deficient mice had impaired IL-18 production mainly from the intestinal epithelial compartment further diminishing the capacity of these mice to recover from colitis. Likewise, overt inflammation and lack of IL-18 in the *Nlrp6*^−/−^ mice has been associated with increased colonic tumor development (216), however, as seen for *Nlrp3*^−/−^ mice it is still unknown whether administration of IL-18 is capable of rescuing the susceptibility phenotype. Interestingly, gene expression profiling of colorectal tumors derived from WT and *Nlrp6*^−/−^ mice revealed an increased expression of paracrine factors of the Wnt and NOTCH signaling cascades, underscoring a novel function of NLRP6 in controlling intestinal proliferation (218). Sensing of damaged or dying cells by NLRP6 and NLRP3 inflammasomes has lately been hypothesized to prevent CRC through maintaining the balance between IL-22 and IL-22 binding protein (IL22-BP) (219). It has been speculated that sensing of DAMPs by both NLRs instigates IL-18-dependent down-regulation of the inhibitory molecule IL-22BP, thus allowing IL-22 to repair the injured tissue. However, dysregulated NLRP6 or NLRP3 signaling could potentially lead to inappropriate IL-22BP expression, thus creating a pro-tumorigenic environment caused by either excessive cell proliferation or lack of tissue repair (219). Although the dual function of IL-22 in CRC has been well-described, further experimental validation is needed to pinpoint the exact mode by which NLRP3 or NLRP6 regulate IL-22/IL-22BP ratio during colon tumorigenesis.

NLRP12 was originally defined as an inflammasome NLR due to its co-localization with ASC and caspase-1, induction of IL-1β and IL-18 secretion as well as NF-κB activation (220, 221). SNPs within the *NLRP12* gene have been associated with increased susceptibility to atopic dermatitis and periodic fever syndromes accompanied mostly with caspase-1 activation and IL-1β release (222–225). It has been observed that NLRP12 can negatively regulate both canonical and non-canonical NF-κB pathways by targeting the IL-1R-associated kinase 1 (IRAK1) and NF-κB inducing kinase (NIK) for proteasomal degradation (226–228). Two independent studies proposed that NLRP12 acts as a tumor suppressive molecule *ex vivo* and in *in vivo* animal models of colitis and colitis-induced CRC (229, 230). Mice lacking NLRP12 have been found to be more susceptible to DSS-injury with increased body weight loss, enhanced pathology scores coupled with massive infiltration of inflammatory cells and high inflammatory cytokine production (229, 230). Furthermore, AOM–DSS treatment of *Nlrp12*^−/−^ mice has been shown to further provoke colonic tumor development and progression (229, 230). In the first study, it was clearly demonstrated that lack of NLRP12 increases NIK-dependent non-canonical NF-κB signaling and drives the regulation of cancer promoting genes like CXCL12 and CXCL13 (230). In the second report, the enhanced tumorigenicity in knockout mice was traced to excessive canonical NF-κB activation due to lack of NLRP12 in hematopoietic cells. Indeed, enhanced LPS-induced canonical NF-κB activation was exhibited in *Nlrp12*^−/−^ macrophages *ex vivo*, suggesting that microbial sensing and negative regulation of inflammation may account for NLRP12-mediated tumor suppression (229). Altogether, these results underscore the importance of anti-inflammatory signals provided by NLRP12 in maintaining colonic homeostasis and protecting from colitis and colon tumorigenesis.

## Therapeutic Strategies and Conclusion

It has been suggested that the strong immunomodulatory properties of NLRs could be exploited for mounting potent anti-tumorigenic responses. In fact, mice injected with B16 melanoma cells or EL4 thymoma cells expressing flagellin from *Salmonella typhimurium* were shown to display dramatic resistance to tumor establishment in NLRC4 dependent manner (231). In addition, immunization with flagellin expressing cancer cells lead to impressive antigen-specific CD4 and CD8 T cell responses via NLRC4 and NAIP5 signaling and bestowed anti-tumor immunity against a secondary inoculation with tumor cells (231). Similarly, activation of NODs, in particular NOD2, to elicit robust cell-based anti-tumor immunity has been under scrutiny for several years. Indeed, instillation of MDP in patients with lung cancer has been reported to enhance expression of inflammatory cytokines and neutrophils in the pleural fluid (232). Relatedly, it has been suggested that the local immune-modulatory activity of MDP helps improve prognosis in hamsters suffering from osteosarcoma (233) and significantly reduces tumor metastasis in several murine cancer models, such as B16–BL6 melanoma, colon 26-M#1 carcinoma, and L5178Y-ML25T T lymphoma (234, 235).

Overt activation of the NLRP3 inflammasome has been demonstrated to elicit cancer progression. For instance, in mouse models of methylcholanthrene (MCA, a highly potent carcinogen) induced fibrosarcoma, NLRP3 was demonstrated to promote cancer progression. Moreover, NLRP3 expression in myeloid cells was shown to interfere with the suppression of cancer metastasis by inhibiting recruitment of anti-tumor NK cells to the site of carcinogenesis (236). Besides interfering with natural tumor control, NLRP3 inflammasome-mediated IL-1β has been described to attenuate anti-tumor effects of chemotherapeutic agents, gemcitabine (Gem), and 5-fluorouracil (5FU) (237). Mice lacking NLRP3 were far more receptive to thymoma regression upon treatment with Gem or 5FU as compared to WT mice. Furthermore, enhanced NLRP3-driven IL-1β release was linked with the induction of T helper 17 (Th17) cells that promoted chemo-resistance in WT mice (237). Keeping these observations in view, several studies support the use of specific inhibitors, antagonists, and monoclonal antibodies against components of the inflammasome, e.g., caspase-1, IL-1β, and IL-18, as therapeutic approaches beneficial for controlling inflammation and improving cancer prognosis (238).

An early phase clinical study suggests that administration of the IL-1R antagonist, Anakinra, alone or in combination with dexamethasone could potentially impede human multiple myeloma progression (239). Furthermore, it was demonstrated that IL-18 derived from tumor cells had the ability to subvert the NK cell-mediated tumor immunosurveillance and to promote tumor progression in a programed death receptor 1 (PD1)-dependent manner (240, 241). These findings suggest the potential of using IL-18 as well as PD1 neutralization for cancer immunotherapy. Overall, selective attenuation of the activities of certain NLRs could potentially boost regression and improve responsiveness to chemotherapy. The variability in NLRP3- and IL-18-mediated effects in different cancers highlights the complexity in NLR circuits and suggests that any broad implications regarding NLR intervention in tumorigenesis should be carefully investigated.

Microbial environment, diet, mouse strain, tumor ontogeny, etc. are all part of the complex network that dictates how an NLR influences inflammation and tumorigenesis. Sensitivity to these factors has lead to conflicting disease phenotypes in genetically modified mice lacking specific NLRs. Furthermore, NLR expression in hematopoietic or non-hematopoietic cellular compartments appears to have distinct influence on inflammatory regulation and tumorigenesis. Due to such discrepancies, it is still uncertain how dysregulation of these innate immune sensors incites inflammation that leads to carcinogenic transformation of cells. Although several mechanisms have been suggested like control of NF-κB signaling, regulation of tissue repair factors, and IL-18 secretion, no unifying hypothesis exists. In addition, interaction of NLRs with different members of the TNFR pathway, BCL2 family of proteins, IAPs, apoptotic caspases, and autophagy regulators point toward more intricate mechanisms for NLR regulation than currently acknowledged. Future studies focusing on the biochemistry of interactions between cell death regulators and NLRs are required to delineate the co-integration of NLR-cell death mechanisms so as to facilitate implementation of NLR modifying therapeutic strategies for inflammatory diseases and cancer.

## Conflict of Interest Statement

The authors declare that the research was conducted in the absence of any commercial or financial relationships that could be construed as a potential conflict of interest.
